# Utilizing High Resolution Satellite Imagery for Automated Road Infrastructure Safety Assessments

**DOI:** 10.3390/s23094405

**Published:** 2023-04-30

**Authors:** Ivan Brkić, Marko Ševrović, Damir Medak, Mario Miler

**Affiliations:** 1 Chair of Geoinformatics, Faculty of Geodesy, University of Zagreb, Kačićeva 26, 10000 Zagreb, Croatia; dmedak@geof.hr (D.M.); mmiler@geof.hr (M.M.); 2 Department of Transport Planning, Faculty of Transport and Traffic Sciences, University of Zagreb, Vukelićeva 4, 10000 Zagreb, Croatia; msevrovic@fpz.unizg.hr

**Keywords:** road assessment, road markings, road safety, satellite imagery

## Abstract

The European Commission (EC) has published a European Union (EU) Road Safety Framework for the period 2021 to 2030 to reduce road fatalities. In addition, the EC with the EU Directive 2019/1936 requires a much more detailed recording of road attributes. Therefore, automatic detection of school routes, four classes of crosswalks, and divided carriageways were performed in this paper. The study integrated satellite imagery as a data source and the Yolo object detector. The satellite Pleiades Neo 3 with a spatial resolution of 0.3 m was used as the source for the satellite images. In addition, the study was divided into three phases: vector processing, satellite imagery processing, and training and evaluation of the You Only Look Once (Yolo) object detector. The training process was performed on 1951 images with 2515 samples, while the evaluation was performed on 651 images with 862 samples. For school zones and divided carriageways, this study achieved accuracies of 0.988 and 0.950, respectively. For crosswalks, this study also achieved similar or better results than similar work, with accuracies ranging from 0.957 to 0.988. The study also provided the standard performance measure for object recognition, mean average precision (mAP), as well as the values for the confusion matrix, precision, recall, and f1 score for each class as benchmark values for future studies.

## 1. Introduction

According to the annual statistical report of the European Safety Road Observatory (ESRO) [1], there were 42 road fatalities per million inhabitants in the European Union (EU) in 2020, while the number of road fatalities was 67 in 2010. The ESRO pedestrian thematic report also informs that 20% of all traffic fatalities are pedestrians, with this percentage increasing to 38% in urban areas, a percentage that has been stable from 2010 to 2018 [2]. The European Commission (EC) has published an EU Road Safety Framework for the period 2021 to 2030 to reduce the above figures [3]. It is a set of intermediate targets to be achieved by 2030 to reach the long-term goal of zero road fatalities by 2050. One of the interim targets is to reduce traffic fatalities by 50% between 2021 and 2030 [3]. To achieve the stated goal, the EC defines Key Performance Indicators (KPIs) to measure progress toward the goals. Road infrastructure and environment are key factors for 30% of road crashes [4]. One of the KPIs therefore relates to road infrastructure and is defined as the percentage of distance traveled on roads with a safety rating above an agreed threshold [3]. Until the methodology and the threshold for safety rating are established, the KPI is defined as the percentage of distance traveled on roads with opposing traffic separation (by barriers or surfaces) relative to the total distance traveled [3]. Currently, the development of the methodology for safety ranking methodology is left to the EU member states. The EC is developing a common methodology based on the Road Infrastructure Safety Management (RISM) Directive 2008/96 and its amendment form directive 2019/1936, wherein indicated infrastructure elements used for road infrastructure safety assessments were defined [5]. While some EU member states have developed their own road assessment methodologies, many EU member states rely on the European Road Assessment Programme (EuroRAP), a European non-profit organization of automobile clubs, road authorities, and researchers [3]. This program results in safety ratings for roads between 1 and 5 stars, with 1-star roads having a high likelihood of road accidents resulting in serious injury or fatality, while 5-star roads have no likelihood of fatal outcomes in accidents [6]. EuroRAP is also a part of the International Road Assessment Programme (iRAP). Currently, iRAP (and therefore EuroRAP) is based on the collection of road and roadside attributes to provide a road safety rating. There are 66 defined road attributes and their classes, which are defined in detail in the iRAP coding manual [7]. Stated attributes can be divided into those which are collected from terrain data (52 attributes) and post-coding attributes (14 attributes).

Considering all the above, there is an obvious need to collect road attributes; they are a crucial factor in determining the level of road safety according to iRAP and in accordance with the EU directive 2019/1936 [5]. Currently, iRAP-defined road attributes are collected manually from georeferenced video [8]. Lately, the development of technology enables using machine and deep learning techniques for collecting road attributes from different sources such as Unmanned Aerial Vehicles (UAVs), georeferenced video (front-view video from vehicles), Light Detection and Ranging (lidar), etc. UAVs are used for traffic flow analysis [9,10,11,12], including number of vehicles’ estimation on inspected roads, which is also one of the iRAP road attributes. In addition, UAVs are used for road marking detection [13,14], as well as road surface distress detection [15]. Vehicle-mounted georeferenced videos are a source of data mostly for traffic sign detection [16,17,18], but also for roadside feature detection [19,20,21,22] and road marking detection [23,24]. Lidar collects high numbers of spatial points as well as other attributes such as color, intensity, number of returns, etc. This makes it suitable for collecting a wide range of road attributes such as roadside feature detection [25,26] road surface distress detection [27], traffic sign detection [28], intersection detection [29], and determination of road geometry characteristics such as slope and curvature [30,31,32]. In terms of satellite imagery and road attributes, most research are focused on road extraction from optical as well as Synthetic-Aperture Radar (SAR) sensors [33,34,35]. They can be used in road attribute collecting processes but are not part of the road attributes defined by iRAP. There are studies that are focused on the direct detection of road attributes from satellite imagery such as road intersection detection [36] and pedestrian crossings [37,38,39].

This study proposed a new approach for road attribute detection using Very High Resolution (VHR) satellite imagery combined with deep learning techniques for object detection. The study focused on the detection of different pedestrian crossing types. Object detection was performed on spatially transformed road segments to distinguish between pedestrian crossings on inspected roads and on side roads. All detected classes of pedestrian crossings are defined in the iRAP Coding Manual [7]. In addition, apart from pedestrian crossings, object detection was performed to determine whether the road was divided or undivided, i.e., to detect objects or areas between carriageways where the carriageways were divided. Finally, school areas were also detected as part of iRAP-defined attributes.

This research has several scientific advantages over existing studies that focus on road attributes detection. First, satellite imagery allows easy global data access and road attribute detection without a physical presence on the road, which is a significant advantage over UAVs, georeferenced video, and lidar. This is especially important for linear objects such as roads, which are difficult to cover with a UAV, but also very expensive with mobile lidar or vehicle-mounted georeferenced video.

The second contribution of this research is the direct harmonization of the detected object classes with the road attributes defined by iRAP without the need for post-processing and reclassification.

The third contribution of this research concerns providing detection of divided and undivided carriageways, which directly relates to the preliminary definition of road infrastructure KPIs from the EC. In short, the detection of these objects allows the evaluation of road safety at this time, even if the methodology and thresholds for road safety are not yet defined. This is also one of the main attributes of a road, as many other attributes are defined differently depending on whether the carriageways are divided or not.

This paper is structured as follows: after a brief introduction of the research topic, the mention of the main contributions of this paper, and a brief overview of recent related studies, the proposed approach is described in detail. The approach is divided into three parts: vector data processing, satellite imagery processing and detection of school zones, pedestrian crossings, and divided carriageways. For a better understanding of the whole process, the framework is presented with a corresponding diagram. Every part of the framework has subsections that describe each step of the proposed approach in detail. This is followed by a results section, which presents the results of the object detection process. The results are presented in the form of tables and confusion matrices. This is followed by a discussion of the results and the main advantages and disadvantages of the approach in comparison to related works. Finally, based on the results and discussion, a brief conclusion is given, indicating future research options to improve the determination of iRAP attributes.

### Related Works

Regarding the use of satellite imagery to detect road attributes, there are several works that focus on road markings and pedestrian crossings. Prakash et al. (2015) [37] proposed a framework for road markings detection. The method was based on satellite imagery from GeoEye and WorldView-2 satellites with a spatial resolution of 0.4–0.5 m. Open Street Map (OSM) data were used as a source of intersection locations. After extracting image tiles with intersections, the images were rotated to align the driving axis on the road with the vertical axis of the images. Finally, a periodic analysis was performed to decide whether a pixel represented an intersection or not. Overall, the recall rate and precision in the test sections were 63% and 89%, respectively. Ahmetovic et al. (2017) [40] proposed a two-stage framework for pedestrian crossing detection. In the first stage, crossing candidates are detected on satellite images provided by Google Map API. Crossings are detected by ZebraLocalizer, an algorithm previously developed by the authors based on the geometric attributes of pedestrian crossings. This algorithm was implemented with a high recognition rate, so the process of generating crossing candidates contained many false positive examples. In the second phase, at the location of the intersection candidate, the authors developed a method for accessing Google Street View panoramic images. This was used for the final detection of whether the pedestrian crossing was at that location or not. The proposed approach achieved 77% to 95% of precision, while the achieved recall ranged from 90.2% to 97.1%. Berriel et al. (2017) [39] proposed a method for pedestrian crossing detection from satellite images. The method consisted of two stages: automatic data acquisition and annotation; model training and classification. OSM data were used to acquire crossing locations, while Google Maps API was used to access satellite imagery. Over 245,000 image tiles with positive and negative examples of crossings from more than 20 cities were created. ConvNet (Convolutional Neural Network) was used for binary image classification and achieved 97.11% accuracy. The spatial resolution of the satellite images used in this study was not reported. Ghilardi et al. (2018) [38] proposed a method to assist visually impaired people. The method was based on satellite imagery provided by Google Map API and a mobile application that warned people of nearby pedestrian crossings. The spatial resolution of the satellite images used was ~0.13 m. Crossing detection was performed using Google Maps road tiles (which are used to extract roads and mask the environment). Then, an SVM classifier was used for binary classification. The generated dataset consisted of 370 images with pedestrian crossings and 570 images without crossings and achieved 94.6% accuracy. Chen et al. (2021) [41] proposed a method for pedestrian crossing detection by fusing object detection tasks and image segmentation tasks. Image segmentation was performed using a U-net structure of CNN with the goal of extracting roads. The segmented images were used in combination with object detection techniques such as You Only Look Once v3 (YOLO v3), Faster-RCNN, and YOLO v3 based on DenseNet 121 to locate crossings. DenseNet-based YOLO v3 achieved the highest accuracy of 94.61%. Satellite images for London suburbs with a spatial resolution of 0.15 m were used in this study, but the source of the images was not provided.

## 2. Materials and Methods

**Figure 1:**
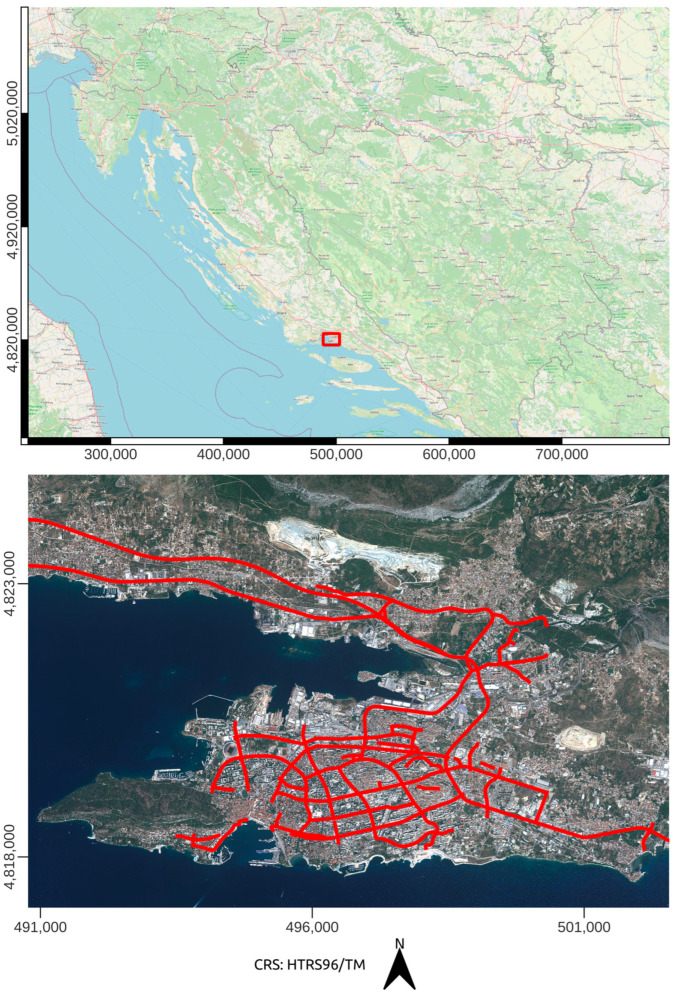
Study area on Open Street Map (OSM) and satellite imagery used in this study with observed roads plotted with red line (created by QuantumGIS software v3.22).

This study was conducted in the wide area of Split. It is the second largest city in Croatia, with 161,312 inhabitants [42]. Therefore, this study focused on the collection of specific road attributes in urban and suburban areas. The total length of roads and highways surveyed was 83.5 km. The study area is shown in Figure 1, with the observed roads marked.

**Figure 2:**
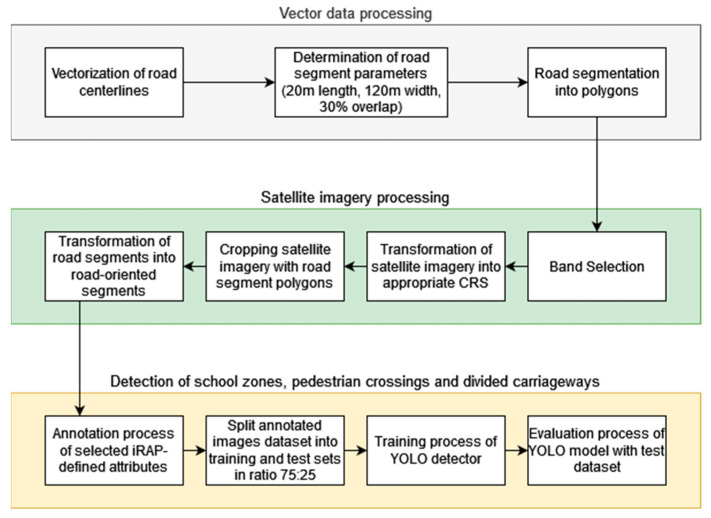
Three-stage workflow for detection of school zones, pedestrian crossings, and physically divided carriageways.

The study was divided into three phases. The first phase consisted of vector data processing, vectorization of road centerlines, and segmentation of observed roads with specific dimensions of road segments and percentage of overlap. The second phase included satellite image processing, band selection, cropping of the images with the boundaries of the road segments, and transformation of the cropped images to align the road centerline with the Y axis of the coordinate reference system (CRS), i.e., generating road-oriented images. Finally, the road segment images were used to detect school zones, physically divided roadways, and four types of pedestrian crossings. A complete overview of the workflow is shown in Figure 2.

### 2.1. Vector Data Processing

The centerlines of all roads and highways were vectorized manually. According to iRAP Manual Coding [7], the road assessment is based on individual roads. Furthermore, the input datum for the assessment of a single road is the centerline, which must be vectorized manually. The single road that is the subject of the assessment is called the inspected road [7]. The process of vectorizing centerlines must comply with the rules established by iRAP. Parts of the inspected road where the carriageways are divided for a length of more than 400 m in a row are coded separately, which means that the centerlines for both carriageways must be vectorized. This role can also be clearly explained by the following equation:

(1) $$nc=\left\{\begin{array}{c} 1,\;\;if\;l\left(x\right)\leq 400m,\\ 2,\;otherwise. \end{array}\right.$$

where *nc* is the number of centerlines to be vectorized and *l*(*x*) is the length of the undivided road sequence.

In this work, all observed roads were vectorized with a single centerline, since one of the results was to detect whether the road was divided or not. After vectorizing the centerlines of the observed roads, each road was divided into 20 m long and 120 m wide segments with 30% overlap. Although the iRAP coding manual defines a 100 m road segment as the basic unit for road evaluation, smaller dimensions were used in this work to facilitate the fitting of the segment images into the YOLO network. With respect to the iRAP coding process, the conversion of smaller segments into 100 m segments was described in our previously published work [25]. When there were several different types of the same attribute, the riskiest attribute was coded. Since this study focused on capturing road attributes in urban and suburban areas, there were roads with different widths. To address this issue, segments were cut with a width of 120 m (60 m on the left and 60 m on the right side of the centerline of the road). In this way, both narrow urban roads and wide highways were included in the segments. An overlap of 30% was performed to avoid losing road attributes collected at the boundaries of the segments. Finally, 5846 road segments were created. The process of creating road segments was performed using the Python programming language and spatial vector-based packages such as GeoPandas and Shapely, while the centerlines were vectorized manually using QuantumGIS. The vectorization process was performed in the Croatian national CRS.

### 2.2. Satellite Imagery Processing

**Figure 3:**
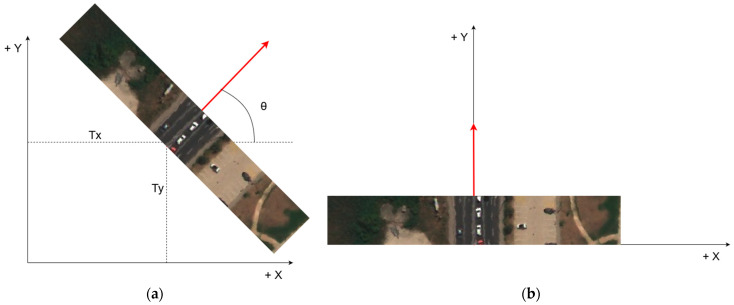
Transformation process of cropped road segments. Red arrow presents centerline of road, while *Tx*, *Ty*, and θ are transformation parameters; (**a**) translation by *Tx* and *Ty* distances and rotation for θ angle; (**b**) transformed road segment with centerline aligned with *Y*-axis (road-oriented).

After preparing the vector data, where the road segments were polygons, the satellite images were processed. The source of the satellite images was the Pleiades Neo 3 satellite launched by Airbus Defence and Space in 2021. The satellite observes every point on Earth twice a day, which allows frequent temporal analysis of objects on Earth, including roads. The date used in this study to acquire the satellite images was 18 August 2022 and the satellite images covered 146.87 km^2^. The spatial resolution of the Pleiades Neo 3 images was 30 cm, while the spectral resolution included seven spectral channels (panchromatic, deep blue, blue, green, red, and near infrared) [43]. In this study, the visible spectral bands were used (blue, green, and red). The satellite images were acquired from the WGS84/UTM zone 33N CRS. The first step was to convert the image position from the source CRS to the Croatian national CRS. Then, the imagery was cropped with the polygons of the created road segments. After cropping, the road segments were transformed into road-oriented segments. The transformation process included translation and rotation operations in the horizontal plane. This can be explained by the equation:

(2) $$\left[\begin{array}{c} x^{\prime }\\ y^{\prime }\\ 1 \end{array}\right]=\left[\begin{array}{ccc} \cos\theta & -\sin\theta & Tx\\ \sin\theta & \cos\theta & Ty\\ 0 & 0 & 1 \end{array}\right]\left[\begin{array}{c} x\\ y\\ 1 \end{array}\right]\;$$

where vector [*x*′, *y*′,1] represents the coordinates of the point in the road segment after the transformation, *Tx* represents the translation in the *x*-axis direction, *Ty* represents the translation in the *y*-axis direction, θ represents the rotation angle, and the vector [*x*, *y*, 1] represents the coordinates of the point in the road segment before the transformation. The transformation process is shown in Figure 3. Finally, to fit the YOLO network, road segment images were converted from a GeoTIFF 16-bit format into a JPEG 8-bit format.

### 2.3. Detection of School Zones, Pedestrian Crossings, and Divided Carriageways

After the transformation of the road segments, an annotation process was performed to create an object detection dataset. To annotate objects, it is necessary to define them clearly. In this study, the focus was on school zone road markings, pedestrian crossings, and divided carriageways. All these attributes are defined by the iRAP Coding Manual [7].

A school zone attribute is divided into four types: school zone area without warnings, marked with road markings or appropriate speed limit signs, school with flashing beacons and appropriate speed limit signs, and areas without school zone. For every road segment, one of the above types must be coded. In this study, school zone areas were annotated with road marking types using satellite imagery. 

**Figure 4:**
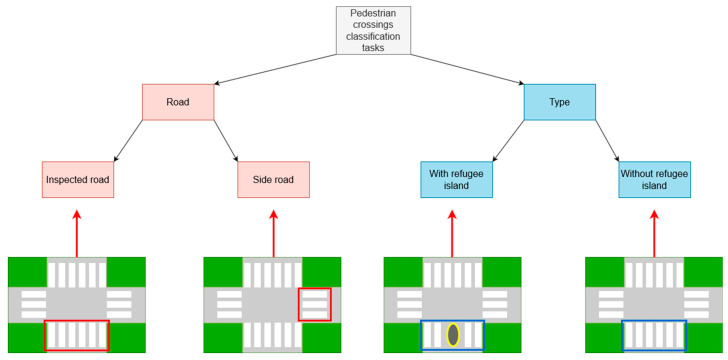
Diagram of pedestrian crossing classification tasks and classes annotated for this study with appropriate examples. Red arrow presents centerline of inspected road.

Pedestrian crossing attributes presented most detected objects. According to the iRAP Coding Manual [7], pedestrian crossings can be divided into two classification tasks. The first classification task refers to whether the pedestrian crossing is on the inspected road or on a side road. This can be distinguished after the processing of vector data and satellite imagery, where each road segment is converted into a road-oriented segment. In addition, the second classification task involves the classification into 11 classes related to the presence of pedestrian crossings, refugee islands, speed bumps, etc. All these classes are defined and described in detail in the iRAP Coding Manual [7]. In this study, two of these classes were found. The first class was the marked pedestrian crossing, which was defined as a clearly marked crossing without a refugee island. The second class was a pedestrian crossing with a refugee island. A refugee island is defined as a purpose-built safe stopping point for pedestrians at the halfway point. It must provide adequate space and protection from passing vehicles and must be seen by drivers. For a better understanding of the pedestrian crossing classification tasks and annotated classes, a diagram of the classification tasks and annotated classes with corresponding examples is shown in Figure 4. 

**Figure 5:**
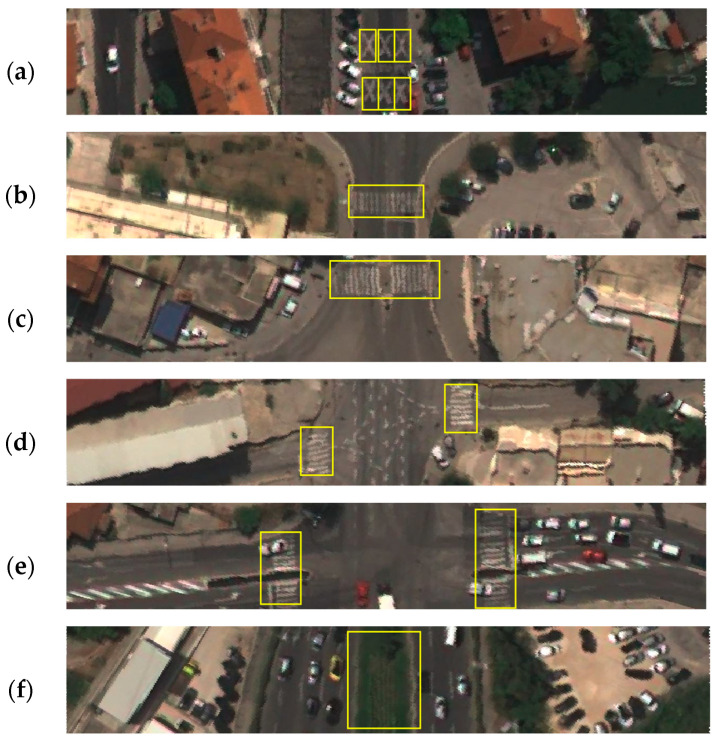
Examples of annotated classes on road segments. (**a**) school zone; (**b**) inspected road pedestrian crossing; (**c**) inspected road pedestrian crossing with refugee island; (**d**) side road pedestrian crossing; (**e**) side road pedestrian crossing with refugee island; (**f**) divided carriageways.

An annotation example of school zone road markings is shown in Figure 5a. By integrating two pedestrian crossing classification tasks, this paper ultimately focused on four classes of pedestrian crossings: pedestrian crossing on the inspected road (Figure 5b), pedestrian crossing on the inspected road with a refugee island (Figure 5c), pedestrian crossing on the side road (Figure 5d), and pedestrian crossing on the side road with a refugee island (Figure 5e).

The final attribute in this study was divided carriageways. While an undivided carriageway has no physical separation between opposing traffic flows, divided carriageways are those that physically separate opposing traffic flows by either a barrier or a wide physical median [7]. To tackle this attribute, divided objects (safety barriers, land areas, etc.) were annotated. An example of a road segment with divided carriageways is shown in Figure 5f.

#### Experiment Analysis

After the annotation process, the YOLO object detector was trained and tested. YOLO is a widely used algorithm. It has a small architecture size and a high inference speed [44]. It is also a single-stage detector with unique features such as small models with respectable inference times [45]. In this work, the fifth version of the YOLO detector was used [46] due to its high inference speed, which was significant for the processing time of a large number of road kilometers. To achieve more accurate models, dataset size can be crucial [47]. After a detailed analysis of existing freely available object detection datasets, no dataset containing iRAP-defined attributes was found. Therefore, a manual annotation was performed using the software LabelImg [48]. All 5846 road segments were annotated and only those that had one of the defined attributes were selected for the learning process. Therefore, 2602 images were selected for the learning process. All images were divided into training and test datasets in a 75:25 ratio of annotated samples. In terms of images, it amounted to 1951 training images and 651 test images. The training process was performed in 600 epochs and 12 h and 30 min on an NVIDIA GeForce RTX 2080 Ti GPU. In addition, the training process included image augmentation to increase the training dataset in order to achieve higher performances. The augmentation process included the transformation of images into Hue, Saturation, Value space (HSV) and left–right and up–down flipping and scaling. The prediction process provided the class of the detected object, the confidence rate (which indicated the probability that the detected object actually belonged to the detected class), and the image coordinates of the bounding boxes of the detected objects.

The evaluation process of the trained YOLO detector is expressed by the mean average precision (mAP), which is a standard for the evaluation of object detection models [49]. It is defined as the mean over classes of the interpolated Average Precision (AP). AP is given by the area under the precision–recall curve of the detected objects [50]. Definitions of precision and recall values are provided in [51]. In the mAP calculation, it was necessary to define what was a true prediction and what was a false prediction. For this purpose, the Intersection over Union (IoU) value had to be defined. The IoU value expressed the ratio between the intersection and union area of the true and predicted bounding box. The IoU value can be explained by the following equation:

(3) $$IoU\left(GT,\;P\right)\frac{\left|GT\cap P\right|}{\left|GT\cup P\right|}$$

where *GT* is a bounding box of the ground truth object, while *P* is a bounding box of the predicted object.

The *IoU* was set to 0.5. Therefore, predicted bounding boxes with an *IoU* greater than 0.5 were considered correct predictions, while others were considered incorrect. If there were multiple correct predictions for the same ground truth object, the predicted bounding box with the highest confidence rate was considered a correct prediction, while the other predictions were classified as incorrect. Therefore, *IoU* was the basic value for providing mAP, as well as the confusion matrix, of ground truth and predicted objects and further calculations of other statistical performance measures such as accuracy and f1 score. The above measures are described in detail in [52].

## 3. Results

**Figure 6:**
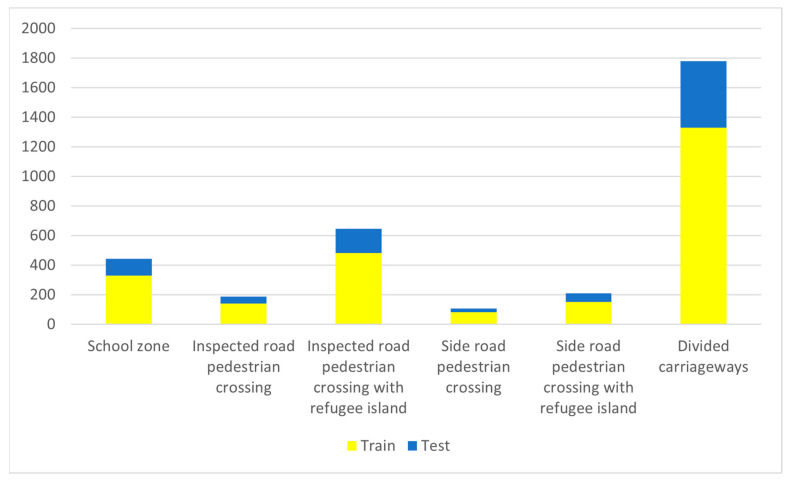
Distribution of annotated samples of school zones, divided carriageways, and pedestrian crossing classes. Number of training samples are shown in yellow, while test samples are shown in blue.

The training dataset contained 2515 samples, while the test dataset contained 862 samples. The distribution of annotated training and test samples is shown in Figure 6. The figure shows more divided carriageway samples in the training and test datasets regarding the other classes.

**Figure 7:**
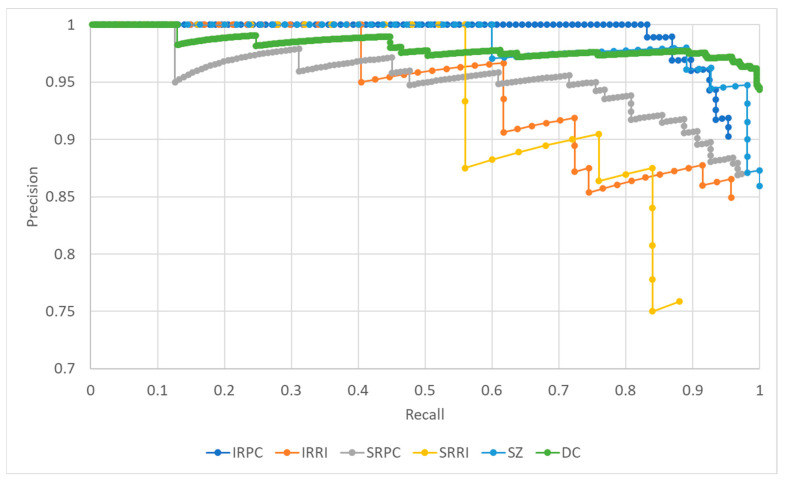
Precision–recall curve generated for each class with IoU and confidence thresholds at 0.5. It is visible that the object detector achieved a significantly lower precision–recall tradeoff for SRRI and IRRI classes.

**Figure 8:**
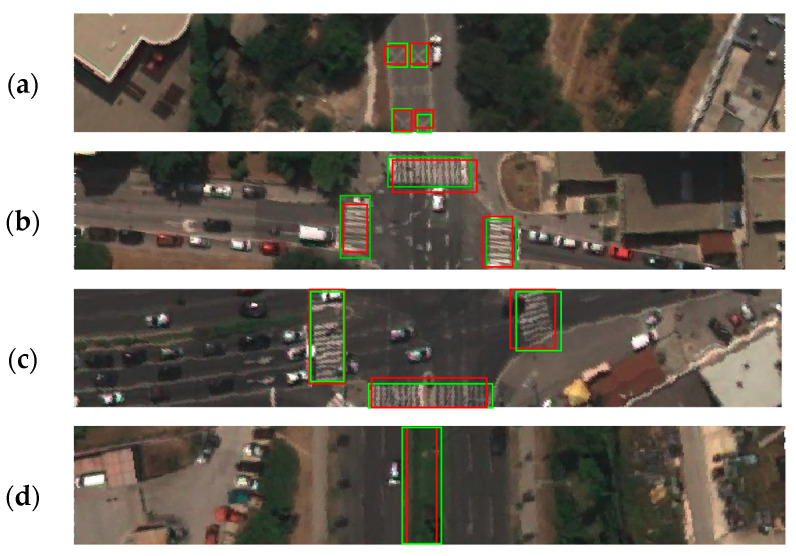
Examples of correctly detected classes by trained YOLO detector. Green bounding boxes show ground truth objects, while red bounding boxes present predicted objects; (**a**) school zone markings; (**b**) inspected and side road pedestrian crossings, (**c**) inspected and side road pedestrian crossings with refugee islands as well as side pedestrian crossing on right side; (**d**) divided carriageways.

**Figure 9:**
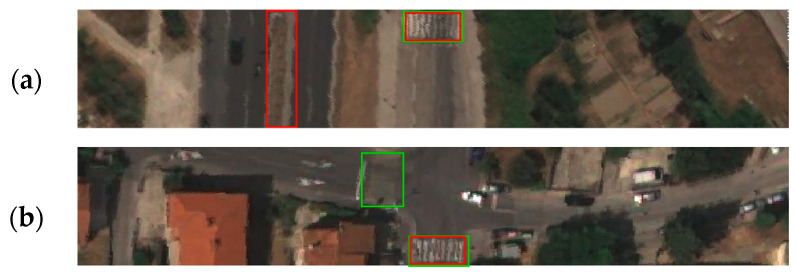

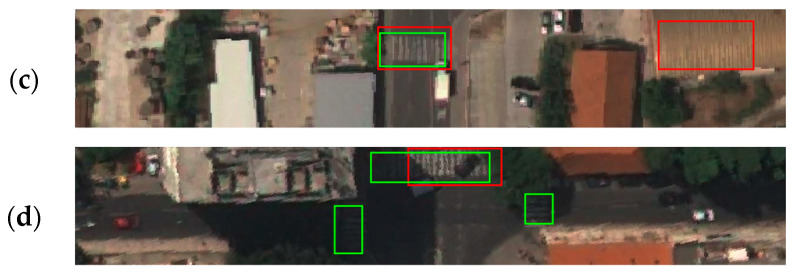
Examples of false positive, false negative, and misleading detections by trained YOLO detector. Green bounding boxes show ground truth objects, while red bounding boxes present predicted objects; (**a**) misleading detection of divided carriageways on non-inspected road; (**b**) false negative detection of side road pedestrian crossing; (**c**) false positive detection of inspected road crossing on roadside object; (**d**) false negative detection of side road pedestrian crossings.

After conducting the training process, the evaluation process resulted in a confusion matrix, which is shown in Table 1. The confusion matrix provided data for the determination of performance measures such as accuracy, precision, recall, and f1 score. The mean values of the stated measures and the mAP value for all classes are shown in Table 2, as well as the same values per class. The precision–recall curve generated for the fixed IoU of 0.5 and confidence of 0.5 is shown in Figure 7. The visualization of correctly detected objects for each class is shown in Figure 8, while examples of false positive, false negative, and misleading detections are shown in Figure 9. Demonstration of detected classes is presented as video in Supplementary Material Video S1.

## 4. Discussion

This study offers significant improvements over related studies. According to EC, there is a need to collect clearly defined road attributes to evaluate road safety. This study focused on collecting school zones, pedestrian crossings, and divided carriageways. All of these road attributes are clearly defined by the iRAP program, which is used in many European countries to assess road safety. As for the detection of school zones, this has not yet been the subject of any research. In this work, it was shown that the integration of satellite imagery with deep learning object detection enabled the detection of iRAP-defined school zones with high efficiency. In addition, divided carriageways have not been the subject of previous studies, although this is a critical attribute for both iRAP- and EC-defined KPI. Road attributes in the iRAP program are defined separately for divided and undivided carriageways. The preliminary KPI definition from the EC also includes divided carriageways. All these indicate that divided carriageway detection will soon be an important step in road safety assessment. In this study, divided carriageway detection was achieved with high efficiency and the performance measures provided could serve as benchmark values for future work.

With respect to pedestrian crossings, there are many works that focus on pedestrian crossing detection from various sources such as UAVs, vehicle-mounted lidars, or georeferenced videos. Considering that high-resolution satellite imagery is a more cost-effective solution, especially for linear objects such as roads that are very difficult and expensive to cover using the aforementioned technologies, this work focused on pedestrian detection using satellite imagery. There are several works that have used satellite imagery for the same task. While related studies have focused on the detection of one type of pedestrian crossings, this study focused on the detection of four iRAP-defined classes of pedestrian crossings; this was a much more detailed but also more challenging task. It was made possible by segmenting roads and transforming segments into road-oriented segments. Therefore, it was easier to distinguish pedestrian crossings on the inspected road from those on the side road.

Compared to related works, Prakash et al. (2015) [37] provided a similar approach in the vector processing of road segments, but they performed a pixel-based periodic analysis on satellite imagery as part of the detection process. They achieved a precision of 0.89 for one class of pedestrian crossings, while the precision in our study ranged from 0.846 to 0.932 for four different types. They also achieved a recall value of 0.63, while our Yolo detector had a recall value between 0.759 and 0.903. In contrast to Prakash et al. (2015) [37], Berriel et al. (2017) [39] performed binary image classification using ConvNet on a large dataset of pedestrian crossing tiles from Google Maps with a binary accuracy of 0.97, while the accuracy in our research ranged from 0.957 to 0.988 for four classes. In addition, Ghilardi et al. (2018) [38] performed classification with an SVM classifier and achieved an accuracy of 0.946, while Chen et al. (2021) [41] used the YOLO v3 deep learning-based detector for pedestrian crossing detection and achieved an accuracy of 0.946. All these performance measures show that our approach had similar efficiency to other deep learning-based approaches such as ConvNet and Yolo v3 and higher efficiency than approaches based on pixel-based periodic analysis and other machine learning algorithms such as SVM classifiers. The results were expected regarding the use of Yolo v5, which has already proven to be better than previous versions of Yolo [53].

Apart from the above advantages and high rate of performance measures, this approach had some limitations. The trained Yolo detector had some disadvantages such as misleading and false detections due to different reasons. With our approach, divided carriageways were detected with high performance measures, but the detected objects were not always on the inspected road. This was a significant problem, especially if the inspected road was an undivided carriageway. Although the detection was correct, it was misleading. This problem could be solved by cutting off less wide road segments that include only a narrow area around the inspected road. In this case, the roads must be divided into different classes to cut off different width road segments depending on the road class. Additionally, one of the major limitations of this approach was that the detection quality was based on the quality of road markings, which depended on road maintenance services. Therefore, we had no control over it. Although there are different laws on adequate road maintenance, unfortunately they are not always realized. This could be overcome with stricter law enforcement. Furthermore, another limitation could be the high rate of false detections due to similar patterns of school zones, pedestrian crossings, and divided carriageways with roadside objects. This is the case when a training dataset is not large enough for the detector to distinguish stated objects. This problem is generally a major obstacle in collecting road attributes based on deep learning approaches. There is no such large dataset that is harmonized with iRAP-defined road attributes. Therefore, it is necessary to build a larger dataset in the future to enable deep learning approaches for road attribute detection. Finally, the major obstacle in urban areas could be unrecognized pedestrian crossings due to shadows, but, with the development of satellite technologies and the annual increase in the number of satellites, this problem could be minimized. More satellites in space could make it possible to avoid shadows by choosing the time of day when the area is observed.

## 5. Conclusions

From the above results and discussion, it is clear that the approach proposed in this paper has several important advantages. First, it focused on the detection of road attributes defined by iRAP, the main framework for road safety assessment in many European countries. Divided carriageway detection is also a significant step forward, as the EC temporarily defines a KPI that includes the length of physically separated roads in its definition. In terms of performance, this approach proved that the integration of satellite imagery and the Yolo object detector achieved a very good performance. The use of high-resolution satellite imagery is a more cost-effective solution, especially for linear objects such as roads. While school zones and divided carriageways have not yet been explored, the performance of detecting pedestrian crossings in four classes could be compared to related work. With an accuracy ranging from 0.957 to 0.988, recall ranging from 0.759 to 0.903, and precision ranging from 0.846 to 0.932, our approach achieved similar or better performances to those in related works. Apart from the above advantages, the approach also had some limitations. The major one was the lack of control over the quality of the road markings. Another obstacle was the lack of an annotated dataset that was harmonized with iRAP attribute definitions. A larger dataset would also lead to fewer false detections. Finally, shadows on satellite imagery could be a serious obstacle for object detection, especially in urban areas.

According to the presented limitations of this research, future research on road attribute detection should include annotations of larger iRAP-harmonized datasets, which would allow greater efficiency of the Yolo detector. It would also be possible to include more spectral bands in the process and to evaluate potential improvements over the three visible bands used in this study. In addition, there are over 60 road attributes defined by iRAP that have not yet been studied for automatic detection. Therefore, there is still much room for the exploration of approaches for the automatic detection of these attributes.

## Figures and Tables

**Table 1:** Confusion matrix of ground truth and predicted objects. In addition to the detected objects, the number of Background False Positive (BFP) and Background False Negative (BFN) detections is given. BFN is given on the predicted axis, while BFP is given on the ground truth axis. Bright shades of green present a lower number of matched classes between ground truth and predicted objects. Contrary, dark shades of green present higher number of matched classes between ground truth and predicted objects.

Ground Truth								
		SZ	IRPC	IRRI	SRPC	SRRI	DC	BFP
**Predicted**	**SZ**	55						9
	**IRPC**		102	1				10
	**IRRI**		5	45				3
	**SRPC**				147	3		19
	**SRRI**			1	4	22		2
	**DC**						433	26
	**BFN**	4	10	1	14	1	21	

SZ—School Zone; IRPC—Inspected Road Pedestrian Crossing; IRRI—Inspected Road pedestrian crossing with Refugee Island; SRPC—Side Road Pedestrian Crossing; SRRI—Side Road pedestrian crossing with Refugee Island; DC—Divided Carriageways; BFP—Background False Positive; BFN—Background False Negative.

**Table 2:** Accuracy, recall, precision, F1 score, and AP for each class as well as mean values of every performance measure.

	Accuracy	Recall	Precision	F1 Score	AP
**SZ**	0.988	0.849	0.938	0.891	0.968
**IRPC**	0.957	0.870	0.891	0.880	0.939
**IRRI**	0.988	0.759	0.846	0.800	0.887
**SRPC**	0.986	0.859	0.932	0.894	0.923
**SRRI**	0.972	0.903	0.872	0.887	0.798
**DC**	0.950	0.943	0.954	0.949	0.979
**Mean**	0.974	0.864	0.905	0.884	0.916

SZ—School Zone; IRPC—Inspected Road Pedestrian Crossing; IRRI—Inspected Road pedestrian crossing with Refugee Island; SRPC—Side Road Pedestrian Crossing; SRRI—Side Road pedestrian crossing with Refugee Island; DC—Divided Carriageways.

## Data Availability

Data available on request due to restrictions. The data presented in this study are available on request from the corresponding author. The data are not publicly available due to the privacy of provided satellite imagery.

## Supplementary Materials

The following supporting information can be downloaded at: https://www.mdpi.com/article/10.3390/s23094405/s1. Video S1: Sequence of road-oriented images in video format demonstrating detected classes on high resolution satellite imagery. Red color—inspected road pedestrian crossing (IRPC); Blue color—school zones (SZ); Purple color—divided carriageway; Cyan color—side road pedestrian crossing with refugee island (SRRI); Green color—side road pedestrian crossing (SRPC); Yellow color—inspected road with refugee island (IRRI)
